# Genetic diversity of BoLA-DRB3 in South American Zebu cattle populations

**DOI:** 10.1186/s12863-018-0618-7

**Published:** 2018-05-22

**Authors:** Shin-nosuke Takeshima, Claudia Corbi-Botto, Guillermo Giovambattista, Yoko Aida

**Affiliations:** 1 Nanomedical Engineering Laboratory, RIKEN Cluster for Pioneering Research, 2-1 Hirosawa, Wako, Saitama, 351-0198 Japan; 2 Viral Infectious Diseases Unit, RIKEN, 2-1 Hirosawa, Wako, Saitama, 351-0198 Japan; 3 Graduate school of frontier sciences, The University of Tokyo, 2-1 Hirosawa, Wako, Saitama, 351-0198 Japan; 4 Institute of Agriculture, Tokyo University of agriculture and technology, 2-1 Hirosawa, Wako, Saitama, 351-0198 Japan; 5 Department of global agricultural science, The University of Tokyo, 2-1 Hirosawa, Wako, Saitama, 351-0198 Japan; 6 Department of Food and Nutrition Faculty of Human Life, Jumonji University, 2-1-28 Sugasawa, Niiza, Saitama, 352-8510 Japan; 7 IGEVET, CCT LA PLATA CONICET, FCV, UNLP, B1900AVW, CC 296 La Plata, Argentina

**Keywords:** BoLA-DRB3, Genetic diversity, Gir, Brahman, Nellore, Sequence-based typing

## Abstract

**Background:**

Bovine leukocyte antigens (BoLAs) are used extensively as markers of disease and immunological traits in cattle. However, until now, characterization of BoLA gene polymorphisms in Zebu breeds using high resolution typing methods has been poor. Here, we used a polymerase chain reaction sequence-based typing (PCR-SBT) method to sequence exon 2 of the BoLA class II DRB3 gene from 421 cattle (116 Bolivian Nellore, 110 Bolivian Gir, and 195 Peruvian Nellore-Brahman). Data from 1416 Taurine and Zebu samples were also included in the analysis.

**Results:**

We identified 46 previously reported alleles and no novel variants. Of note, 1/3 of the alleles were detected only in Zebu cattle. Comparison of the degree of genetic variability at the population and sequence levels with genetic distance in the three above mentioned breeds and nine previously reported breeds revealed that Zebu breeds had a gene diversity score higher than 0.86, a nucleotide diversity score higher than 0.06, and a mean number of pairwise differences greater than 16, being similar to those estimated for other cattle breeds. A neutrality test revealed that only Nellore-Brahman cattle showed the even gene frequency distribution expected under a balanced selection scenario. The F_ST_ index and the exact G test showed significant differences across all cattle populations (F_ST_ = 0.057; *p* <  0.001). Neighbor-joining trees and principal component analysis identified two major clusters: one comprising mainly European Taurine breeds and a second comprising Zebu breeds. This is consistent with the historical and geographical origin of these breeds. Some of these differences may be explained by variation of amino acid motifs at antigen-binding sites.

**Conclusions:**

The results presented herein show that the historical divergence between Taurine and Zebu cattle breeds is a result of origin, selection, and adaptation events, which would explain the observed differences in BoLA-DRB3 gene diversity between the two major bovine types. This allelic information will be important for investigating the relationship between the major histocompatibility complex and disease, and contribute to an ongoing effort to catalog bovine MHC allele frequencies according to breed and location.

**Electronic supplementary material:**

The online version of this article (10.1186/s12863-018-0618-7) contains supplementary material, which is available to authorized users.

## Background

Domestic bovines comprise two major breed types: humpless (*Bos taurus*) and humped (*Bos indicus* or *Bos taurus indicus*). Based on archeological and molecular evidence, it is estimated that the Taurine and Zebu lineages divergence occurred from a common ancestor between 330,000 and 2 million years ago, respectively [1–4]. Since then, the two types of cattle have accumulated many genetic variations resulting in a high genetic distance; this has contributed to highly differentiated phenotypes and adaptation. Currently, Zebu breeds are found worldwide in tropical and subtropical regions; indeed, beef production in South America’s warm regions is based mainly on Zebu cattle (Nellore, Brahman, and Gir breeds).

The bovine MHC, named bovine leukocyte antigen (BoLA), comprises three DRB class II copies, with the BoLA-DRB3 gene being the most highly expressed and polymorphic [5]. Polymorphic sites within the BoLA-DRB3 gene are located mainly at peptide-binding sites; these polymorphisms are mostly maintained by balancing selection [6]. Several lines of evidence show that polymorphisms influence both the magnitude and epitope specificity of antigen-specific T cell responses to infectious diseases [7]. In this sense, the BoLA-DRB3 gene is associated with differences in susceptibility to infectious disease (e.g., bovine leukosis virus-induced lymphocytosis, mastitis, and dermatophilosis), different immunological and production traits (e.g., milk yield), and different vaccine responses (e.g., to foot-and-mouth disease and *Theileria parva*.) [8, 9].

Since the first pioneering studies based on serotype analysis [10, 11], others have used different genotyping techniques to examine the genetic diversity of the BoLA-DRB3 gene in a large number of bovine breeds. Currently, polymerase chain reaction (PCR)-sequence-based typing (SBT) [12–22] and next generation sequencing (NGS) [23] are the most powerful methods. More than 25 years’ work has identified 107 BoLA-DRB3 alleles in cattle; these are listed in the Immuno Polymorphism Database (IPD)–MHC database (https://www.ebi.ac.uk/ipd/mhc/group/BoLA). Forty-two of them were reported in *Bos indicus*.

SBT has better discriminatory power and precision than PCR-RFLP to identify diversity of BoLA-DRB3 alleles in different cattle breeds [15]. However, BoLA-DRB3 diversity has been characterized at the DNA sequence level only in 12 out of over 800 breeds of cattle recognized worldwide [12–23]. Studied breeds include European taurine (e.g., Hereford, Angus, Shorthorn, Holstein, Jersey, Overo Colorado, and Overo Negro), Asian taurine (Japanese Black, Philippine Native, and Korean Native), American Creole (e.g., Yacumeño and Hartón del Valle), and different types of cross-breed (e.g., Holstein × Sahiwal, Philippine Native × Brahman, and Philippine Native × Holstein).

Until now, several private Zebu BoLA-DRB3 alleles have been reported by authors using indirect techniques such as PCR-RFLP, followed by cloning and sequencing [24–27]. These studies focused mainly on screening and analysis of a few animals from some Zebu breeds (e.g., Brahman, Dairy Gir, Boran, and Fulani); however, no study has used SBT or NGS assays to examine genetic diversity of BoLA-DRB3 in large samples. With this in mind, the aim of the present study was to examine the genetic diversity of the BoLA-DRB3 gene in three Zebu breeds from Bolivia and Peru, and to compare the results with those reported for Taurine breeds. The results may contribute to an ongoing effort to catalog bovine MHC allele frequency according to breed and location.

## Methods

### Sample populations and genomic DNA extraction

Blood samples were obtained from 421 Zebu cattle belonging to Bolivian Nellore (*N* = 116), Bolivian Gir (*N* = 110), and Peruvian Nellore-Brahman (*N* = 195) breeds. These breeds were selected because they are bred most widely in South America. In addition, previously reported data from 1416 animals from Taurine and Zebu breeds from Japan, the Philippines, Colombia, Chile, and Bolivia were included for comparison [12–22]. Detailed information about the breeds/populations analyzed is provided in Table 1. Genomic DNA was extracted from 40 μl of whole blood spotted on FTA Elute cards (Whatman, Tokyo, Japan), according to the manufacturer’s instructions.

**Table 1:** Detailed information about the populations analyzed

Acronym	Sample size	Number of farm	Breed	Type	Origin (country)	Sampling country	Reference
NeBo	116	2	Nellore	Zebu	Brazil	Bolivia	Present work
GirBo	110	2	Gir	Zebu	India	Bolivia	Present work
BrPh	236	6	Brahman	Zebu	USA	Philippines	Present work
HeCh	49	2	Hereford	taurine	Great Britain	Chile	Takeshima et al., [17]
ShoJa	100	Random Collection	Japanese Shorthorn	taurine	Japan	Japan	Takeshima et al., [17]
JEJa	69	Random Collection	Japanese Jersey	taurine	Channel Island	Japan	Takeshima et al., [17]
HoJa	102	Random Collection	Japanese Holstein	taurine	Netherlands	Japan	Takeshima et al., [17]
WaJa	200	Random Collection	Japanese Black	taurine	Japan	Japan	Takeshima et al., [17]
HV	66	1	Hartón del Valle	taurine	Colombia	Colombia	Giovambattista et al., [12]
YA	112	4	Yacumeno	taurine	Bolivia	Bolivia	Giovambattista et al., [12]
NaPh	482	35	Philippine Native	taurine	Philippines	Philippines	Takeshima et al., [20]
NexBrPe	195	1	Nellore × Brahman	Zebu mixed		Peru	Present work

Cattle were handled by veterinarians from local farms, RIKEN, and Universidad Autónoma Gabriel René Moreno, in strict accordance with good animal practice following the Universidad Austral de Chile Institutional guidelines. Veterinarian Collaborator explained about the sample collection to farmer in verbal way, and their future use in the research project. This study was approved by Committees on the Ethics of Animals for Research at the National from University of LA PLATA (Argentine: Certificate date May 26th, 2014), Universidad Autónoma Gabriel Moreno (Bolivia, and Universidad Austral de Chile (Certificate No. 153–2014).

### BoLA-DRB3 typing using PCR-sequence based typing (SBT)

BoLA-DRB3 alleles were genotyped using PCR-SBT. Briefly, exon 2 of BoLA-DRB3 was amplified by PCR as described by Takeshima et al. [19], using primers DRB3FRW and DRB3REV [28]. The PCR fragments were purified using an ExoSAP-IT PCR Product Purification Kit (USB Corp., Cleveland, OH) and sequenced using the ABI PRISM BigDye Terminator Cycle Sequencing Ready Reaction Kit (Applied Biosystems, Foster City, CA). Raw sequence data were analyzed using Assign 400ATF ver. 1.0.2.41 software (Conexio Genomics, Fremantle, Australia).

### Statistical analysis

#### Measures of genetic variability

Allele frequencies and the number of alleles (*N*_a_) were obtained by direct counting, and 95% confidence intervals (CI) for allele frequencies were calculated using the binomial distribution implemented in R with the binom.confint function (http://cran.r-project.org/web/packages/binom/). The distribution of alleles across major groups of breeds was represented by a Venn plot using the R package ‘VennDiagram’. The observed (*H*_o_) and expected unbiased (*H*_e_) heterozygosity for the BoLA-DRB3 locus were estimated as described previously [29] using ARLEQUIN 3.5 software for population genetic analyses [30]. Potential deviations from Hardy-Weinberg equation (HWE) were estimated for each breed by *F*_IS_ statistics [31] using the exact test included in GENEPOP 4.0 [32]. The Ewens–Watterson–Slatkin exact test for neutrality was estimated as described by Slatkin [33] and implemented in ARLEQUIN 3.5 software.

### Genetic structure and differentiation

Genetic structure and genetic differentiation among breeds were assessed using Wright’s F statistics (F_ST_) and the previously described variance-based method [31]. These parameters were estimated using ARLEQUIN 3.5 and GENEPOP 4.0. *F*_ST_ values were represented graphically using the pairFstMatrix.r function in the R statistical environment.

### Principal component analysis (PCA)

To condense the genetic variation at the BoLA-DRB3 locus, allele frequencies were used to perform principal component analysis (PCA) as described previously [34] using PAST software [35]. Nei’s standard genetic distance (*D*s) [36] and Nei’s unbiased genetic distance (*D*_*A*_) [37] were calculated from allele frequencies, and cluster analysis was performed using the UPGMA [38] and the neighbor-joining (NJ) [39] algorithms. For these analyses, only pure breeds and a Nellore-Brahman mixed population were included due to limitations inherent in the dendrograms. CIs for the groupings were estimated by bootstrap re-sampling of data using 1000 replications. Genetic distances and trees were calculated using POPULATIONS 1.2.28 software [40], and trees were constructed using TREEVIEW [41].

### Genetic diversity at the nucleotide and amino acid sequence levels

Nucleotide diversity (π) and pairwise comparison of nucleotide substitutions between alleles were calculated using ARLEQUIN 3.5. The mean number of nonsynonymous (dN) and synonymous (dS) nucleotide substitutions per site was estimated for each pair using the Jukes–Cantor’s formula as described by Nei and Gojobori [42]. These parameters were estimated using MEGA 5 [43].

## Results

### Distribution of BoLA-DRB3 alleles among analyzed zebu cattle breeds

**Fig. 1:**
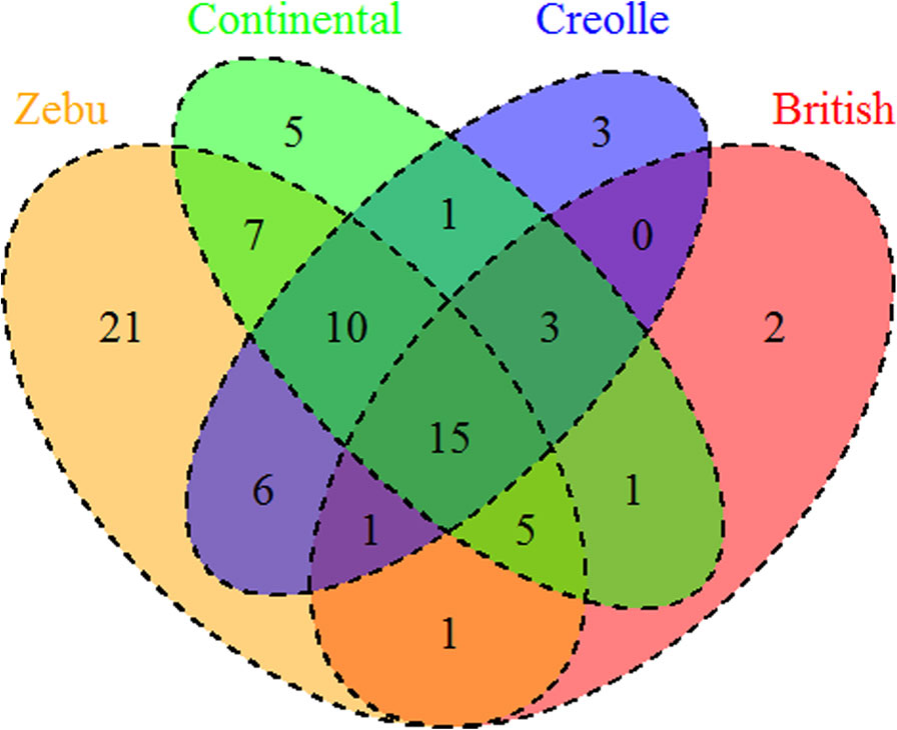
Venn diagram showing BoLA-DRB3 allele distribution among cattle breeds grouped according to geographical origin: British, European Continental, American Creole, and Cebu

PCR-SBT genotyping allowed to identify 46 previously reported BoLA-DRB3 alleles, while no new variants were observed (Table 2) and all genotyping result were shown in additional table (Additional file 1). The *N*_a_ ranged from 19 BoLA-DRB3 alleles in Gir to 33 variants in Nellore-Brahman (Table 2). Of these, six alleles reported in the IPD-MHC public database (https://www.ebi.ac.uk/ipd/mhc/) were from *Bos indicus* and 26 alleles were from *B. taurus*; 14 alleles were shared among these two major bovine types. Furthermore, a Venn diagram was constructed using data obtained in this study and from previous reports [12–22]; these data, that included 14 breeds and cross-breeds, were then used to evaluate the distribution of the BoLA-DRB3 allele (Fig. 1.). To achieve this, data were grouped in terms of geographical origin, i.e., British, European Continental, American Creole, and Zebu cattle breeds. The plot clearly shows that the Zebu group, that comprised Bolivian Gir, Bolivian Nellore, Peruvian Nellore-Brahman and Philippine Brahman breeds, harbors 21 out of the 90 alleles identified in the four cattle groups (Fig. 1); these 21 alleles represent about 30% of the 66 alleles detected in the Zebu group.

**Table 2:** Gene frequencies (%) of BoLA-DRB3 alleles detected by PCR-SBT in Bolivian Nellore, Bolivian Gir, and Peruvian Nellore-Brahman mixed cattle populations

DRB3 Allele	Nellore	Gir	Nellore-Brahman	Bolivian Holstein	Bovine Type	Reference
	(N = 116)	(N = 110)	(N = 195)	(*N* = 102)		
DRB3*0101	0 (0.00–1.58)	0 (0.00–1.66)	0.26 (6.49e^**−** 05^ – 1.42)	5.66 (3.40–8.80)	*Bos taurus*	Mikko & Anderson, [25]
DRB3*0201	0 (0.00–1.58)	3.63 (1.58–7.04)	1.79 – (0.72–3.66)	2.83 (1.30–5.30)	*Bos taurus*	Takeshima et al., [18]
DRB3*03021	0.86 (0.10–3.08)	0 (0.00–1.66)	0.77 (0.16–2.23)	0 (0.00–1.20)	*Bos taurus*	Takeshima et al., [18]
DRB3*0601	0 (0.00–1.58)	0 (0.00–1.66)	0.26 (6.49^e-05^ – 1.42)	10.38 (0.00–2.20)	*Bos taurus*	Mikko & Anderson, [25]
DRB3*0701	0 (0.00–1.58)	0 (0.00–1.66)	1.54 (0.57–3.32)	1.57 (0.50–3.60)	*Bos taurus*	Takeshima et al., [18]
DRB3*0902	1.29 (0.27–3.73)	0.91 (0.11–3.24)	15.90 (12.41–19.91)	15.41 (11.60–19.90)	*Bos indicus* *Bos taurus*	Mikko & Anderson, [25] Takeshima et al., [18]
DRB3*1001	2.59 (0.95–5.54)	0 (0.00–1.66)	0 (0.00–0.94)	10.69 (7.50–14.60)	*Bos Taurus*	Mikko & Anderson, [25]
DRB3*1101	0.43 (0.01–2.38)	0 (0.00–1.66)	0.51 (0.06–1.84)	11.32 (8.10–15.30)	*Bos Taurus*	Takeshima et al., [18]
DRB3*1103	1.29 (0.27–3.73)	0 (0.00–1.66)	0.51 (0.06–1.84)	0 (0.00–1.20)	*Bos indicus*	Maillard et al., [26]
DRB3*1201	0 (0.00–1.58)	0 (0.00–1.66)	0.77 (0.16–2.23)	4.72 (2.70–7.70)	*Bos Taurus*	Mikko & Anderson, [25]
DRB3*1302	2.59 (0.95–5.54)	0 (0.00–1.66)	0 (0.00–0.94)	0 (0.00–1.20)	*Bos Taurus*	Takeshima et al., [18]
DRB3*1501	0 (0.00–1.58)	**15.45 (10.94–20.92)**	1.79 (0.72–3.66)	18.24 (14.20–22.90)	*Bos indicus* *Bos Taurus*	Mikko & Anderson, [25] Takeshima et al., [18]
DRB3*1601	1.72 (0.47–4.35)	0 (0.00–1.66)	0.26 (6.49^e-05^ – 1.42)	0.31 (0.00–1.70)	*Bos Taurus*	Mikko & Anderson, [25]
DRB3*1602	0 (0.00–1.58)	3.18 (1.29–6.45)	0 (0.00–0.94)	0 (0.00–1.20)	*Bos Taurus*	Mikko et al., [56]
DRB3*1701	0 (0.00–1.58)	0 (0.00–1.66)	1.28 0.42–2.97)	1.26 (0.30–3.20)	*Bos Taurus*	Mikko & Anderson, [25]
DRB3*1703	0 (0.00–1.58)	0 (0.00–1.66)	0.51 (0.06–1.84)	0 (0.00–1.20)	*Bos indicus*	Maillard et al., [26]
DRB3*1801	0.43 (0.01–2.38)	0 (0.00–1.66)	0.26 (6.49^e-05^ – 1.42)	3.14 (1.50–5.70)	*Bos Taurus*	Takeshima et al., [18]
DRB3*1901	0 (0.00–1.58)	0 (0.00–1.66)	1.28 0.42–2.97)	0 (0.00–1.20)	*Bos Taurus*	Mikko & Anderson, [25]
DRB3*2003	0 (0.00–1.58)	2.73 (1.01–5.84)	0.77 (0.16–2.23)	0 (0.00–1.20)	*Bos indicus*	Maillard et al., [26]
DRB3*2101	0 (0.00–1.58)	7.73 (4.57–12.08)	0 (0.00–0.94)	0 (0.00–1.20)	*Bos indicus*	Mikko & Anderson, [25]
DRB3*2201	23.28 (17.99–29.25)	7.73 (4.57–12.08)	**32.05 (27.44–36.93)**	0.94 (0.20–2.70)	*Bos indicus* *Bos Taurus*	Mikko & Anderson, [25] Takeshima et al., [17]
DRB3*2601	3.8 (1.79–7.24)	0 (0.00–1.66)	0 (0.00–0.94)	0 (0.00–1.20)	*Bos indicus* *Bos Taurus*	Mikko & Anderson, [25]
DRB3*2703	0 (0.00–1.58)	0 (0.00–1.66)	3.85 (2.17–6.26)	4.72 (2.70–7.70)	*Bos Taurus*	Takeshima et al., [18]
DRB3*2705	0 (0.00–1.58)	0 (0.00–1.66)	0.77 (0.16–2.23)	0 (0.00–1.20)	*Bos indicus* *Bos Taurus*	Maillard et al., [26] Takeshima et al., [18]
DRB3*2710	0.86 (0.10–3.08)	0.45 (0.01–2.51)	7.95 (5.46–11.09)	0 (0.00–1.20)	*Bos Taurus*	Takeshima et al., [17]
DRB3*2801	**24.57 (19.17–30.63)**	7.27 (4.21–11.54)	2.56 (1.24–4.66)	0 (0.00–1.20)	*Bos indicus* *Bos Taurus*	Gelhaus et al., [24] Takeshima et al., [18]
DRB3*2802	1.72 (0.47–4.35)	5.45 (2.85–9.33)	0 (0.00–0.94)	0 (0.00–1.20)	*Bos indicus* *Bos Taurus*	Russell et al., [57] Gelhaus et al., [24] Takeshima et al.,[19]
DRB3*3001	5.17 (2.70–8.86)	0 (0.00–1.66)	2.05 (0.89–4.00)	0.31 (0.00–1.70)	*Bos indicus* *Bos Taurus*	Gelhaus et al., [24] Takeshima et al., [18]
DRB3*3101	7.33 (4.33–11.47)	5.91 (3.18–9.89)	1.79 (0.72–3.66)	0 (0.00–1.20)	*Bos Taurus*	Takeshima et al., [18]
DRB3*3202	1.72 (0.47–4.35)	0.45 (0.01–2.51)	0 (0.00–0.94)	0 (0.00–1.20)	*Bos Taurus*	Takeshima et al., [19]
DRB3*3301	2.16 (0.70–4.96)	1.36 (0.28–3.93)	1.03 (0.28–2.60)	0 (0.00–1.20)	*Bos Taurus*	Sitte et al., [58]
DRB3*3401	0.43 (0.01–2.38)	0 (0.00–1.66)	0 (0.00–0.94)	0 (0.00–1.20)	*Bos Taurus*	Takeshima et al., [18]
DRB3*3501	5.60 (3.02–9.39)	0 (0.00–1.66)	1.54 (0.57–3.32)	0 (0.00–1.20)	*Bos indicus* *Bos Taurus*	Maillard et al., [26] Takeshima et al., [18]
DRB3*3601	2.16 (0.70–4.96)	11.82 (7.87–16.83)	6.15 (3.98–9.02)	1.89 (0.70–4.10)	*Bos indicus* *Bos Taurus*	Maillard et al., [26] Takeshima et al., [18]
DRB3*3701	0 (0.00–1.58)	0 (0.00–1.66)	1.03 (0.28–2.60)	0 (0.00–1.20)	*Bos Taurus*	Takeshima et al., [16]
DRB3*3901	0 (0.00–1.58)	1.82 (0.50–4.59)	0 (0.00–0.94)	0 (0.00–1.20)	*Bos Taurus*	
DRB3*4101	0 (0.00–1.58)	5.00 (2.52–8.77)	0 (0.00–0.94)	0 (0.00–1.20)	*Bos indicus Bos Taurus*	Takeshima et al., [18]
DRB3*4201	0.86 (0.10–3.08)	0 (0.00–1.66)	0.51 (0.06–1.84)	0 (0.00–1.20)	*Bos indicus Bos Taurus*	Maillard et al., [26] Takeshima et al., [18]
DRB3*4301	0.86 (0.10–3.08)	8.64 (5.28–13.16)	0 (0.00–0.94)	0 (0.00–1.20)	*Bos indicus* *Bos taurus*	Maillard et al., [26] Takeshima et al., [18]
DRB3*4302	0 (0.00–1.58)	0 (0.00–1.66)	0 (0.00–0.94)	0 (0.00–1.20)	*Bos taurus*	Wang et al., [59]
DRB3*4401	1.29 (0.27–3.73)	0 (0.00–1.66)	0 (0.00–0.94)	0.31 (0.00–1.70)	*Bos indicus* *Bos taurus*	Maillard et al.,[26] Takeshima et al., [19]
DRB3*4802	2.59 (0.95–5.54)	0 (0.00–1.66)	0 (0.00–0.94)	0 (0.00–1.20)	*Bos taurus*	Takeshima et al., [19]
DRB3*5701	0 (0.00–1.58)	0 (0.00–1.66)	0.51 (0.06–1.84)	0 (0.00–1.20)	*Bos taurus*	Wang et al., [59]
DRB3*5702	3.8 (1.79–7.24)	0.45 (0.01–2.51)	5.13 (3.16–7.81)	0 (0.00–1.20)	*Bos indicus* *Bos taurus*	Das et al., [60] Takeshima et al., [18]
DRB3*14011	0.43 (0.01–2.38)	10.00 (6.37–14.75)	1.54 (0.57–3.32)	1.57 (0.50–3.60)	*Bos taurus*	Mikko et al., [25]
DRB3*20012	0 (0.00–1.58)	0 (0.00–1.66)	0.26 (6.49^e-05^ – 1.42)	0 (0.00–1.20)	*Bos taurus*	Gelhaus et al., [24]
DRB3*25011	0 (0.00–1.58)	0 (0.00–1.66)	2.82 (1.42–4.99)	0 (0.00–1.20)	*Bos indicus* *Bos taurus*	Mikko et al., [25] Takeshima et al., [18]
n_a_	26	19	33	18		

N = number of typed unrelated individuals

n_a_ = number of alleles detected in each population

The most frequent alleles in each breed are presented in bold

Confidence intervals for allele frequencies are shown in brackets

**Fig. 2:**
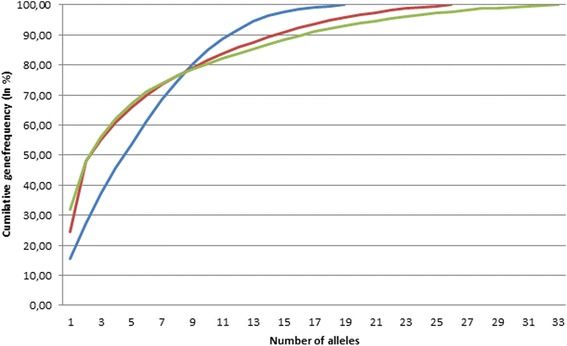
Cumulative gene frequency plot for Bolivian Gir (blue), Bolivian Nellore (red), and Peruvian Nellore × Brahman (green)

The N_a_ within each Zebu population with frequencies > 5% varied; from five in Bolivian Nellore and Peruvian Nellore-Brahman to ten in Bolivian Gir. Some of the high-frequency (> 5%) alleles were present in at least two out of three Zebu populations; these alleles were BoLA-DRB3*2801, *2201, *3101, and *3601 (Table 2). These common alleles account for 65.95% of all alleles sampled in Bolivian Nellore, 67.18% in Peruvian Nellore-Brahman, and 85.00% in Bolivian Gir (Fig. 2).

### Nucleotide and amino acid diversity of BoLA-DRB3 alleles in analyzed zebu breeds

Genetic diversity was examined at the DNA level using two parameters: π and the number of pairwise differences (NPD). The results obtained were then compared with those previously reported for ten other breeds (Table 3). The π ranged from 0.068 in the Nellore × Brahman population to 0.078 in the Bolivian Gir population, while the mean NPD varied from 16.91 in the Nellore × Brahman to 19.43 in Bolivian Gir. The 13 cattle breeds analyzed by PCR-SBT varied in terms of the number and composition of BoLA-DRB3 alleles. However, with the exception of Shorthorn and Native Philippine cattle, the values observed for the π and NPD indices were similar to those estimated for other cattle breeds (Table 3).

**Table 3:** Values for nucleotide diversity (Π), the mean number of pairwise differences (NPD), and the mean number of non-synonymous and synonymous nucleotides substitutions per site

Breed	Π	NPD	Total		ABS	
			ds	dn	ds	dn
Nellore^a^	0.070	17.53	0.037	0.103	0.187	0.319
Gir^a^	0.078	19.43	0.041	0.106	0.183	0.328
Nellore x Brahman^a^	0.068	16.91	0.042	0.108	0.187	0.328
Brahman Philippine^b^	0.085	21.10	0.058	0.116	0.224	0.311
Native Philippine^b^	0.141	33.40	0.052	0.113	0.222	0.306
Yacumeño^c^	0.079	19.78	0.039	0.105	0.151	0.484
Hartón del Valle^c^	0.076	19.00	0.034	0.109	0.140	0.484
Japanese Shorthorn^d^	0.146	36.56	0.047	0.106	0.188	0.504
Japanese Holstein^d^	0.082	20.50	0.092	0.151	0.198	0.520
Japanese Black^d^	0.071	18.59	0.093	0.149	0.174	0.519
Japanese Jersey^d^	0.086	19.20	0.109	0.164	0.175	0.547
Chilean Hereford^e^	0.069	17.39	0.035	0.104	0.161	0.460
Hanwoo^f^	0.072	18.06	0.047	0.111	0.153	0.505

^a^Present work

^b^Takeshima et al., 2014

^c^Giovambattista et al., [12]

^d^Takeshima et al., [17] and Miyasaka et al., 2011

^e^Takeshima et al., [21]

^f^Lee et al., 2011

ds and dn were estimated for the entire sequence and for the antigen biding sites (ABS)

We also calculated the average number for dn and ds in Bolivian Nellore, Peruvian Nellore × Brahman, and Bolivian Gir. As shown in Table 4, the values obtained for these populations (0.037 to 0.042 for ds and 0.103 to 0.108 for dn) when analyzing the whole of exon 2 suggested a dn/ds difference range for this genome region of 0.058 to 0.066. The dn/ds differences were more prominent when only the antigen-binding sites (ABS) were analyzed (dn/ds > 0.132; Table 3).

**Table 4:** Number of alleles (n_a_), observed (h_o_) and expected heterozygosity (h_e_), and Hardy-Weinberg equilibrium (HWE), measured using F_IS_, and Slatkin’s exact neutrality test

Breed	N	n_a_	h_o_	h_e_	F_IS_ - p value	Slatkin’s exact *p* value
Nellore^a^	116	26	0.78	0.87	0.099–0.741	0.271
Gir^a^	110	19	0.88	0.92	0.041–0.153	0.008
Nellore x Brahman^a^	195	33	0.76	0.86	0.113 - < 0.001	0.470
Philippine Brahman^b^	236	58	0.89	0.95	0.069 < 0.001	0.139
Philippine Native^b^	482	71	0.91	0.96	0.048 – < 0.001	0.092
Yacumeño^c^	113	36	0.92	0.95	0.034–0.78	0.006
Hartón del Valle^c^	66	24	0.97	0.94	−0.036 - 0.0004	0.136
Japanese Shorthorn^d^	100	20	0.92	0.91	−0.009 - 0.095	0.061
Japanese Holstein^d^	102	18	0.92	0.90	−0.021 - 0.358	0.083
Japanese Black^d^	200	23	0.90	0.91	0.009–0.362	0.003
Japanese Jersey^d^	69	14	0.91	0.89	−0.030 - 0.0005	0.055
Chilean Hereford^e^	49	15	0.82	0.87	0.057–0.557	0.580
Hanwoo^f^	359	39	ND	0.90	ND	ND

^a^Present work

^b^Takeshima et al., [20]

^c^Giovambattista et al., [12]

^d^Takeshima et al., [17] and Miyasaka et al., [13]

^e^Takeshima et al., [21]

^f^Lee et al., [23]

N = sample size

ND = not determined

### Gene diversity, HWE, and neutrality testing of BoLA-DRB3 in the analyzed zebu populations

Genetic diversity within the three analyzed Zebu populations was estimated according to the *N*_a_ and the observed and expected heterozygosities. As mentioned above, the *N*_a_ ranged from 19 in Bolivian Gir to 33 in Peruvian Nellore-Brahman, while the values of expected heterozygosity ranged from 0.86 in Nellore-Brahman to 0.92 in Bolivian Gir (Table 4). The observed heterozygosity in Peruvian Nellore-Brahman and Bolivian Gir varied from 0.76 to 0.88, respectively (Table 4). Regarding HWE, two out of three populations were in equilibrium, while Nellore × Brahman showed a significant deviation from the theoretical values (Table 4). This deviation can be explained by a significant excess of homozygous animals (F_IS_ = 0.113, *p* value = 0.033). To assess balancing selection, Slatkin’s exact neutrality test was performed (Table 4). The BoLA-DRB3 gene frequency profile in Gir cattle showed an even distribution (*p* = 0.008) despite the finding that its genotype proportions did not deviate from the theoretical proportions. This even gene frequency distribution is consistent with the theoretical proportion expected under recent balancing selection as opposed to positive or neutral selection (*p* > 0.025). Conversely, the Nellore and Nellore × Brahman gene frequency distributions were compatible with neutral selection (Table 4).

### Genetic structure and differentiation of BoLA-DRB3 in zebu breeds

**Fig. 3:**
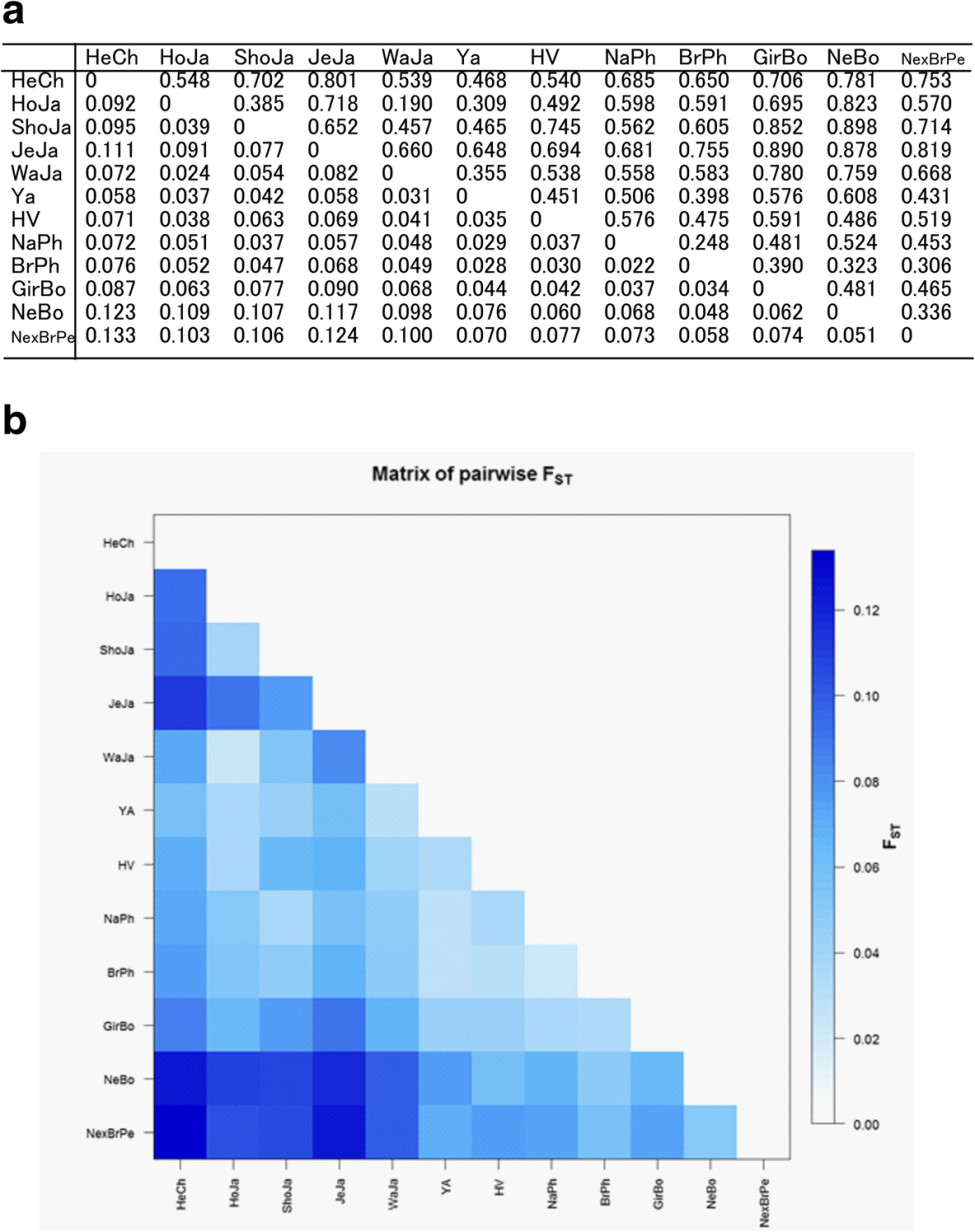
**a** Genetic distance between pairs of populations estimated by Wright’s F statistics (F_ST_) (below) and Nei’s D_A_ distance (above). **b** Graphical representation of calculated F_ST_ values between population pairs using an R-function: pairFstMatrix.r. HeCh = Chilean Hereford; HoJa = Japanese Holstein; ShoJa = Japanese Shorthorn; JeJa = Japanese Jersey; WaJa = Japanese Black; Ya = Yacumeño; HV = Hartón del Valle; NaPh = Philippine Native; BrPh = Philippine Brahman; GirBo = Bolivian Gir; NeBo = Bolivian Nellore; and NexBrPe = Peruvian Nellore × Brahman

To examine population genetic structure and differentiation levels among cattle breeds, we calculated two parameters: the F_ST_ index and the exact G test. There were significant differences in the F_ST_ parameter across all cattle populations (F_ST_ = 0.057; *p* value ≤0.0001); pairwise comparison ranged from 0.022 (between Philippine Brahman and Philippine Native) to 0.133 (between Peruvian Nellore × Brahman and Chilean Hereford) (Fig. 3). However, there was around 5% differentiation among Zebu populations. The exact G test for differentiation indicated that, on average, differences in allele frequency distribution among populations were highly significant (exact p value ≤0.0001). Furthermore, when all pairs of breeds were tested, significant results (p value ≤0.0001) were also obtained for all comparisons, indicating a significant level of genetic structure for BoLA-DRB3 among breeds. In conclusion, when Zebu breeds were included in the analysis, the average F_ST_ value was 1 to 5% higher than that obtained when analyzing taurine breeds alone [12] or a population of the same breeds [22], respectively.

### Genetic differentiation of BoLA-DRB3 alleles in zebu and taurine breeds

**Fig. 4:**
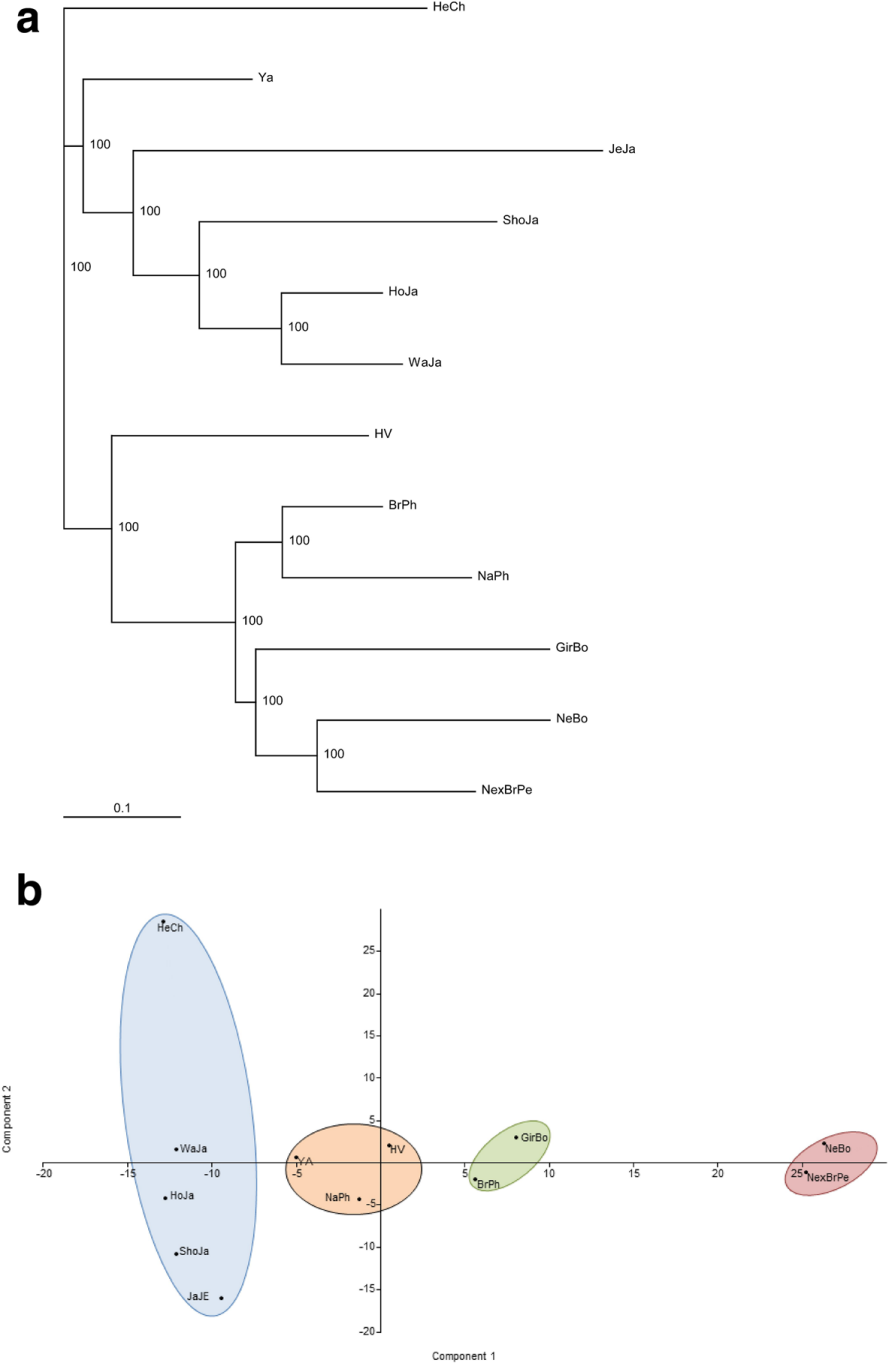
**a** Neighbor-joining tree constructed from a matrix of Nei’s D_A_ genetic distances. **b** Principal component analysis of 12 cattle breeds based on BoLA-DRB3 allele frequencies. HeCh = Chilean Hereford; WaJa = Japanese Black; HoJa = Japanese Holstein; ShoJa = Japanese Shorthorn; JaJe = Japanese Jersey; YA = Yacumeño; NaPh = Native Philippine; HV = Hartón del Valle; BrPh = Philippine Brahman; GirBo = Bolivian Gir; NexBrPe = Peruvian Nellore × Brahman; and NeBo = Bolivian Nellore

To assess the genetic differentiation of BoLA-DRB3 alleles between Zebu cattle and nine breeds previously studied by SBT, we performed two types of analysis: dendrograms and PCA. First, BoLA-DRB3 allele frequencies were used to generate Nei’s D_A_ and D_S_ genetic distances matrices; data from Bolivian Nellore, Bolivian Gir, Peruvian Brahman × Nellore, and nine previously reported breeds (Japanese Jersey, Japanese Shorthorn, Japanese Holstein, Japanese Black, Chilean Hereford, Philippine Native, Philippine Brahman, Yacumeño, and Hartón del Valle; [12–22]) were used. Dendrograms based on distance were constructed using UPGMA and NJ algorithms. NJ cluster analysis based on Nei’ D_A_ or D_S_ genetic distances showed two major clusters: one comprising mainly European taurine breeds and Yacumeño Creole cattle and one comprising Zebu breeds, Hartón del Valle, and Philippine Native breeds (Fig. 4a). Japanese Jersey or Chilean Hereford was located outside of these clusters. UPGMA cluster analysis showed a similar trend, with Jersey and Hereford breeds located in separated clades (data not shown).

Second, we used allele frequencies of BoLA-DRB3 from the 12 breeds mentioned above to perform PCA. The results are reported in Fig. 4b, which shows the first and second principal components (PCs) for BoLA-DRB3 gene frequency. The first two components accounted cumulatively for 44.68% of the variability in the data. The first PC accounted for 28.34% of the total variance, and showed a clear differentiation pattern between Zebu (positive values) and Taurine breeds (negative values), whereas native breeds were located near the origin of the plot. The first PC was determined mainly by 26 alleles, nine with a positive value (BoLA-DRB3*0902, *2201, *2710, *2801, *3001, *3101, *3501, *3601, and *5702) and 17 with a negative value (BoLA-DRB3*0101, *0201, *0301, *0701, *0801, *1001, *1101, *1201, *1501 *1601, *1801, *2006, *2502, *3202, *4501, *14011, and *20012). It is noteworthy that almost all alleles with higher negative values for PC1 were previously reported in *B. Taurus* in the MHC diversity database, whereas the variants that account for PC1 positive values were reported in both *B. Taurus* and/or *B. indicus*. The second PC explained 16.34% of the total variation and shows a gradient among taurine breeds, with Hereford and Jersey located at opposite ends. The second PC was determined mainly by differences in the frequency of 20 alleles; seven with a positive value (BoLA-DRB3*1002, *1501, *1601, *1801, *3202, *14011, and *20012) and 13 with a negative value (BoLA-DRB3*0101, *0201, *0301, *0501, *0701, *0801, *1101, *1201, *2006, *2502, *3701, *4401, and *4501). Finally, the third PC accounted for 14.95% of the variance, similar to PC2; this component allowed differentiation of the *B. taurus* breed, but with Holstein and Jersey at opposite ends. These PCA results are in agreement with the overall clustering observed after NJ or UPGMA tree construction. Native breeds occupy an intermediate position in both analyses.

**Fig. 5:**
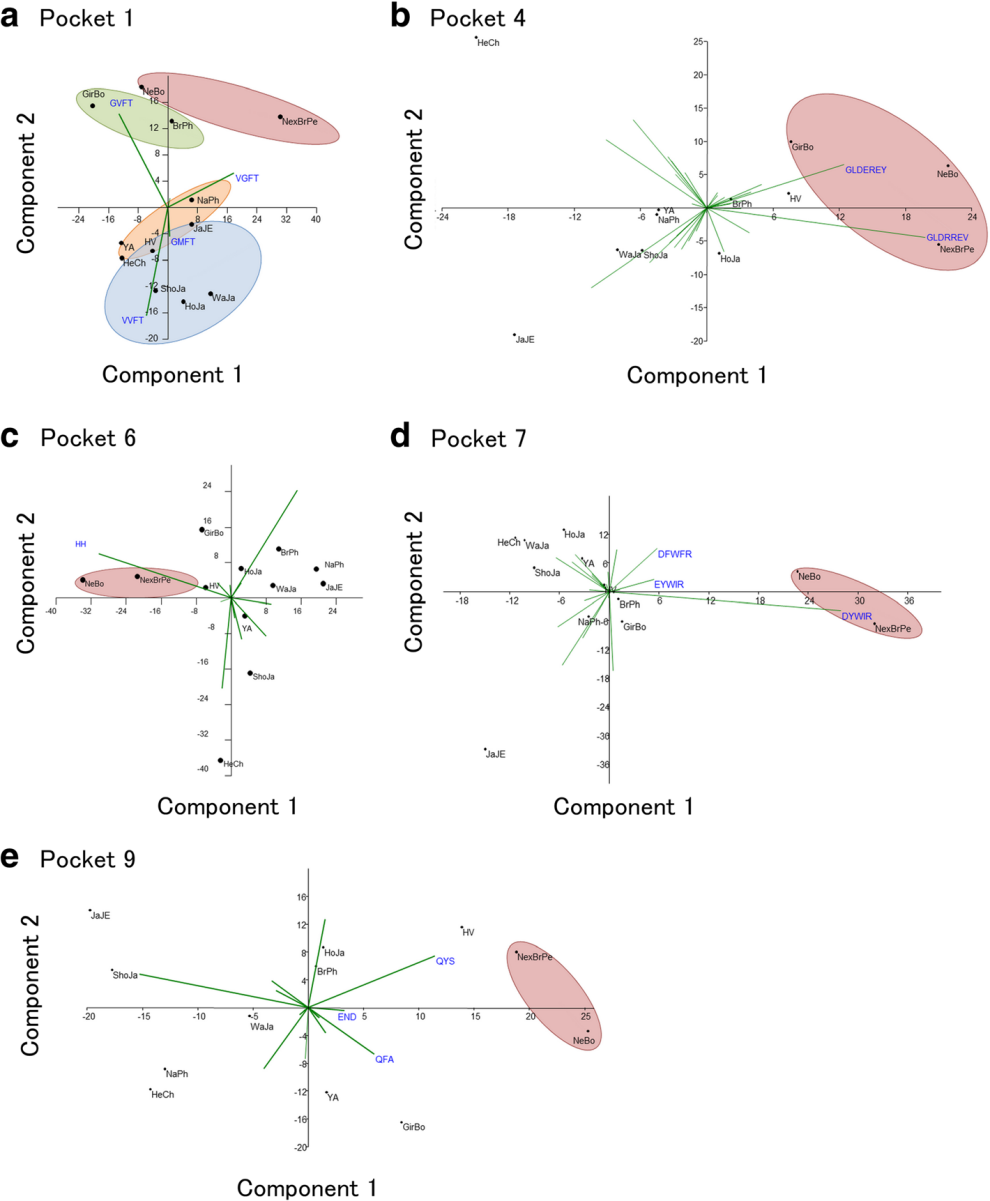
Principal component analysis of 12 cattle breeds based on the amino acid motif form antigen binding site (ABS) of BoLA-DRB3. **a** Pocket 1. **b** Pocket 4. **c** Pocket 6. **d** Pocket 7. **e** Pocket 9. HeCh = Chilean Hereford; WaJa = Japanese Black; HoJa = Japanese Holstein; ShoJa = Japanese Shorthorn; JaJe = Japanese Jersey; YA = Yacumeño; NaPh = Native Philippine; HV = Hartón del Valle; BrPh = Philippine Brahman; GirBo = Bolivian Gir; NexBrPe = Peruvian Nellore × Brahman; and NeBo = Bolivian Nellore. G,Glycine;P,Proline;A,Alanine;V,Valine;L,Leucine;I,Isoleucine;M,Methionine;F,Phenylalanine;Y,Tyrosine;W,Tryptophan;H,Histidine;R,Arginine;Q,Glutamine;N,Asparagine;E,Glutamic Acid;D,Aspartic Acid;S,Serine;T,Threonine

BoLA class II molecule binds various peptides derived from antigens via five antigen binding pockets named pocket 1, pocket 4, pocket 6, pocket 7 and pocket 9 [44]. To assess whether observed differences in allelic frequency between Taurine and Zebu populations is reflected within amino acid motifs in each pocket, we analyzed protein pockets implicated in antigen-binding function by PCA. With respect to taurine breeds, Zebu breeds were clearly differentiated in component 2 of pocket 1 due to a higher frequency of GVFT and VGFT and a lower frequency of VVFT and GMFT amino acid motifs (Fig. 5). Component 1 of pockets 4 and 7 allowed differentiation of Zebu (positive values) from Taurine breeds (negative values). GLDEREY and GLDRREV amino acid motifs in pocket 4, and DYWIR, DFWFR, and EYWIR amino acid motifs in pocket 7, provided the major contribution and explain the distribution observed in PCA plots. With the exception of Philippine Brahman, Zebu breeds showed a similar trend in pocket 9, which was accounted for by QYS, QFA, and END amino acid motifs. Finally, component 1 of pocket 6 only discriminated Nellore from Nellore crossbred by means of the HH motif.

## Discussion

Domestic cattle were derived from the now-extinct wild aurochs (*Bos primigenius*) [45]. Zooarcheological evidence revealed that this wild species was widespread throughout Eurasia and North Africa, even though, events of bovine domestication occurred only few times in the human history and in precise geographic regions (called domestication center). In this sense, different local wild auroch subspecies populations would have been domesticated in five domestication centers: three for *B. taurus* (southwest Asia, north Africa, and northeastern China) at about 10,000 Years B.P., and two for *B. indicus* (Indus Valley and southern India) at around 8000 Years B.P. Mitochondrial DNA (mtDNA) has been extensively used to reconstruct the ancestry and domestic history of cattle. Analysis of modern and ancient mtDNAs showed that auroch diversity had an uneven distribution with multiple mtDNA clades and a complex branching structure [46, 47]. Since domestication process occurred in geographically restricted areas, only a fraction of the whole wild genetic variation was retained in each domestication center (sampling effect). Furthermore, domestic cattle mtDNA phylogenies exhibited two major distinct clades, including taurine T1, T2, T3 and T4, and Zebu I1 and I2 haplogroups [1].

Regarding MHC Class II genes, trans-species theory supports the hypothesis that MHC alleles preceded the origin of related species and survived through the speciation process [48]. Phylogenetic analysis of Bovidae DRB sequences also supports this theory [49, 50]. Bearing this in mind, it is expected that domestic cattle BoLA-DRB3 diversity was present in the auroch population before the divergence of major bovine types. Just as mtDNA haplogroups, BoLA-DRB3 alleles could have shown an uneven distribution in the *B. primigenius* population at the time of domestication, keeping only a fraction of the total diversity in each cattle lineage. Likewise, a later bottleneck having occurred during the more recent breed formation may have increased the genetic differentiation at BoLA-DRB3 alleles. On the other hand, genetic structure could have been diluted due to migration and gene introgression events occurred since domestication. Most of the detected alleles in Zebu were reported in the IPD-MHC database as Taurine, while a slightly smaller number of variants was shared among these two major bovine types, and only six were described as *B. indicus*. In contrast, Venn diagram, used to evaluate the distribution of the BoLA-DRB3 allele among group of breeds, clearly shows that the Zebu group, comprised of Bolivian Gir, Bolivian Nellore, Peruvian Nellore-Brahman and Philippine Brahman breeds, harbors 30% of Zebu privative alleles. This data further supports the hypothesis that BoLA-DRB3 diversity has an uneven distribution.

MHC class II gene diversity can be maintained by some form of balancing or overdominant selection [51, 52]. To assess this hypothesis, allele diversity, gene frequency profile, HWE and Slatkin’s exact neutrality tests were performed. In this sense, between 19 and 33 alleles were detected within the analyzed Zebu breeds, resulting in values of expected heterozygosity higher than 0.85. Furthermore, common alleles accounted for 65.95% of all alleles sampled in Bolivian Nellore, 67.18% in Peruvian Nellore-Brahman, and 85.00% in Bolivian Gir. Despite the fact Zebu breeds exhibit higher resistance to tropical pathogens; these results demonstrate that analyzed populations showed allele diversity and gene frequency distribution within the range reported for Taurine breeds [12, 13, 22].

Regarding HWE, none of the analyzed populations showed a significant excess of heterozygotes animals that could reflect overdominant selection, while only Gir gene frequency profile showed an even distribution consistent with the theoretical proportion expected under recent balancing selection. Even though this evolutionary force could not be detected in most cattle populations studied until now [12, 22], this even gene distribution was observed in Yacumeño and Japanese Black breeds. Furthermore, several of the analyzed breeds showed neutrality test *p* values ranging from 0.025 to 0.1 which, though not significant, could reflect the same bias towards balancing selection. Conversely, Nellore and Nellore × Brahman gene frequency distributions were compatible with neutral selection.

Observed diversity at allele level was also present when analyzing the nucleotide and amino acid variability. The 13 cattle breeds analyzed by PCR-SBT varied in terms of the number and composition of BoLA-DRB3 alleles. However, with the exception of Shorthorn and Native Philippine cattle, the values observed in Zebu breeds for the π and NPD indices were similar to those estimated for other cattle breeds [12, 20–22]. As expected, the dn/ds differences were more prominent when only the ABS were analyzed, which is in concordance with the biological function of these sites. Of note, the average number for dn and ds across the entire exon 2, as well as the ABS, in Zebu breeds was slightly smaller than the average reported for previously analyzed breeds [12, 20–22].

Genetic structure and differentiation of BoLA-DRB3 in Zebu breeds were assessed by calculating F_ST_ index and the exact G test. Both estimations revealed significant differences across all cattle populations. Noteworthy, when Zebu breeds were included in the analysis, the average F_ST_ value was 1 to 5% higher than that obtained when analyzing taurine breeds alone [12] or populations of the same Taurine breed [22], respectively. Moreover, dendrograms based on Nei’ D_A_ or D_S_ genetic distance were constructed using UPGMA and NJ algorithms. These trees showed similar relationship between breeds: two major clusters revealing topologies consistent with the historical and geographical origin. In addition, PCA results are in agreement with the overall clustering observed after NJ or UPGMA tree construction, which showed a clear divergence between Taurine and Zebu populations. Also, native breeds occupy an intermediate position in both analyses, probably due to some level of Zebu gene introgression [53, 54]. Finally, both dendrograms and PCA showed that Chilean Hereford and Japanese Jersey were the most divergent populations among *B. taurus* breeds. This topology could be partially explained by the ancient genetic structure present among and within *B. primigenius* sub-species; and the sampling effect during the domestication process. Furthermore, subsequent selection, local adaptation and breed origin could also have contributed to the observed genetic differentiation at BoLA-DRB3 gene.

PCA based on allelic frequencies of amino acid motif of pockets implicated in antigen-binding function showed a clear differentiation between Taurine and Zebu breeds, as consequence of an enrichment of particular amino acid motif in specific pocket. These differences could be consistent with the widely reported Zebu cattle high disease tolerance/susceptibility to tropical infectious disease, such as ticks and intestinal parasites [47, 55]. Further studies involving structural modeling and molecular simulation of the BoLA-DRβ protein are needed to elucidate whether these differences play a role in the function of the resultant protein.

## Conclusion

The present study is the first to use PCR-SBT to examine the allelic distribution of the BoLA-DRB3 gene in Zebu populations. The results show that the historical divergence between Taurine and Zebu cattle breeds is due to origin, selection, and adaptation events, which explains the observed differences in BoLA-DRB3 gene diversity between these two major bovine types.

## Additional file

Additional file 1: Information and genotyping data used in this study. (XLSX 173 kb)

## Abbreviations

A: Alanine
BoLAs: Bovine leukocyte antigens
CI: confidence intervals
D: Aspartic Acid
D_A_: Nei’s unbiased genetic distance
dN: nonsynonymous
Ds: Nei’s standard genetic distance
dS: synonymous
E: Glutamic Acid
F: Phenylalanine
FST: Wright’s F statistics
G: Glycine
H: Histidine
He: expected unbiased heterozygosity
Ho: observed heterozygosity
HWE: Hardy-Weinberg equilibrium
I: Isoleucine
IPD: Immuno Polymorphism Database
L: Leucine
M: Methionine
N: Asparagine
Na: number of alleles
NGS: next generation sequencing
NPD: number of pairwise differences
P: Proline
PCA: principal component analysis
PCR: polymerase chain reaction
PCR-SBT: Polymerase chain reaction sequence-based typing
PCs: principal components
Q: Glutamine
R: Arginine
S: Serine
T: Threonine
T: Tyrosine
V: Valine
W: Tryptophan
π: Nucleotide diversity

## References

[1] Achilli A, Olivieri A, Pellecchia M, Uboldi C, Colli L, Al-Zahery N, Accetturo M, Pala M, Kashani BH, Perego UA (2008). Mitochondrial genomes of extinct aurochs survive in domestic cattle. Curr Biol.

[2] Bradley D, Loftus R, Cunningham P, MacHugh D (1998). Genetics and domestic cattle origins. Evol Anthropol.

[3] Hiendleder S, Lewalski H, Janke A (2008). Complete mitochondrial genomes of Bos taurus and Bos indicus provide new insights into intraspecies variation, taxonomy and domestication. Cytogenet Genome Res.

[4] Loftus RT, MacHugh DE, Bradley DG, Sharp PM, Cunningham P (1994). Evidence for two independent domestications of cattle. Proc Natl Acad Sci U S A.

[5] Aida Y (1995). Characterization and expression of bovine MHC class II genes. Bull Soc Fr Jpn Sci Vet.

[6] Satta Y, Ohuigin C, Takahata N, Klein J (1994). Intensity of natural-selection at the major histocompatibility complex loci. Proc Natl Acad Sci U S A.

[7] Xu A, van Eijk MJ, Park C, Lewin HA (1993). Polymorphism in BoLA-DRB3 exon 2 correlates with resistance to persistent lymphocytosis caused by bovine leukemia virus. J Immunol.

[8] Takeshima SN, Aida Y (2006). Structure, function and disease susceptibility of the bovine major histocompatibility complex. Anim Sci J.

[9] Nayeri S, Stothard P (2016). Tissues, metabolic pathways and genes of key importance in lactating dairy cattle. Springer Science Reviews.

[10] Davies CJ, Andersson L, Joosten I, Mariani P, Gasbarre LC, Hensen EJ (1992). Characterization of bovine MHC class II polymorphism using three typing methods: serology, RFLP and IEF. Eur J Immunogenet.

[11] Davies CJ, Joosten I, Andersson L, Arriens MA, Bernoco D, Bissumbhar B, Byrns G, van Eijk MJ, Kristensen B, Lewin HA (1994). Polymorphism of bovine MHC class II genes. Joint report of the fifth international bovine lymphocyte antigen (BoLA) workshop, Interlaken, Switzerland, 1 august 1992. Eur J Immunogenet.

[12] Giovambattista G, Takeshima SN, Ripoli MV, Matsumoto Y, Franco LA, Saito H, Onuma M, Aida Y (2013). Characterization of bovine MHC DRB3 diversity in Latin American creole cattle breeds. Gene.

[13] Miyasaka T, Takeshima SN, Matsumoto Y, Kobayashi N, Matsuhashi T, Miyazaki Y, Tanabe Y, Ishibashi K, Sentsui H, Aida Y (2011). The diversity of bovine MHC class II DRB3 and DQA1 alleles in different herds of Japanese black and Holstein cattle in Japan. Gene.

[14] Miyasaka T, Takeshima SN, Sentsui H, Aida Y (2012). Identification and diversity of bovine major histocompatibility complex class II haplotypes in Japanese black and Holstein cattle in Japan. J Dairy Sci.

[15] Takeshima S, Ikegami M, Morita M, Nakai Y, Aida Y (2001). Identification of new cattle BoLA-DRB3 alleles by sequence-based typing. Immunogenetics.

[16] Takeshima S, Nakai Y, Ohta M, Aida Y (2002). Short communication: characterization of DRB3 alleles in the MHC of Japanese shorthorn cattle by polymerase chain reaction-sequence-based typing. J Dairy Sci.

[17] Takeshima S, Saitou N, Morita M, Inoko H, Aida Y (2003). The diversity of bovine MHC class II DRB3 genes in Japanese black, Japanese shorthorn, Jersey and Holstein cattle in Japan. Gene.

[18] Takeshima SN, Matsumoto Y, Aida Y (2009). Short communication: establishment of a new polymerase chain reaction-sequence-based typing method for genotyping cattle major histocompatibility complex class II DRB3. J Dairy Sci.

[19] Takeshima SN, Matsumoto Y, Miyasaka T, Arainga-Ramirez M, Saito H, Onuma M, Aida Y (2011). A new method for typing bovine major histocompatibility complex class II DRB3 alleles by combining two established PCR sequence-based techniques. Tissue Antigens.

[20] Takeshima SN, Miyasaka T, Polat M, Kikuya M, Matsumoto Y, Mingala CN, Villanueva MA, Salces AJ, Onuma M, Aida Y (2014). The great diversity of major histocompatibility complex class II genes in Philippine native cattle. Meta Gene.

[21] Takeshima SN, Miyasaka T, Matsumoto Y, Xue G, Diaz Vde L, Rogberg-Munoz A, Giovambattista G, Ortiz M, Oltra J, Kanemaki M (2015). Assessment of biodiversity in Chilean cattle using the distribution of major histocompatibility complex class II BoLA-DRB3 allele. Tissue Antigens.

[22] Takeshima SN, Giovambattista G, Okimoto N, Matsumoto Y, Rogberg-Munoz A, Acosta TJ, Onuma M, Aida Y (2015). Characterization of bovine MHC class II DRB3 diversity in South American Holstein cattle populations. Tissue Antigens.

[23] Lee BY, Hur TY, Jung YH, Kim H (2012). Identification of BoLA-DRB3.2 alleles in Korean native cattle (Hanwoo) and Holstein populations using a next generation sequencer. Anim Genet.

[24] Gelhaus A, Schnittger L, Mehlitz D, Horstmann RD, Meyer CG (1995). Sequence and PCR-RFLP analysis of 14 novel BoLA-DRB3 alleles. Anim Genet.

[25] Mikko S, Andersson L (1995). Extensive MHC class II DRB3 diversity in African and European cattle. Immunogenetics.

[26] Maillard JC, Renard C, Chardon P, Chantal I, Bensaid A (1999). Characterization of 18 new BoLA-DRB3 alleles. Anim Genet.

[27] da Mota AF, Martinez ML, Coutinho LL (2004). Genotyping BoLA-DRB3 alleles in Brazilian dairy Gir cattle (Bos indicus) by temperature-gradient gel electrophoresis (TGGE) and direct sequencing. Eur J Immunogenet.

[28] Baxter R, Hastings N, Law A, Glass EJ (2008). A rapid and robust sequence-based genotyping method for BoLA-DRB3 alleles in large numbers of heterozygous cattle. Anim Genet.

[29] Nei M (1978). Estimation of average heterozygosity and genetic distance from a small number of individuals. Genetics.

[30] Schneider S, Roessli D, Excoffier L (2000). Arlequin version 2.000: a software for population genetics data analysis. 2.000 edn: genetics and biometry laboratory.

[31] Weir B, Cockerham C (1984). Estimating F-statistics for the analysis of population structure. Evolution.

[32] Rousset F (2008). genepop'007: a complete re-implementation of the genepop software for windows and Linux. Mol Ecol Resour.

[33] Slatkin M (1996). A correction to the exact test based on the Ewens sampling distribution. Genet Res.

[34] Cavalli-Sforza LL, Menozzi P, Piazza A (1994). The history and geography of human genes.

[35] Hammer Ø, Harper DAT, Ryan PD (2001). PAST: paleontological statistics software package for education and data analysis. Palaeontol Electron.

[36] Nei M (1972). Genetic distance between populations. Am Nat.

[37] Nei M, Tajima F, Tateno Y (1983). Accuracy of estimated phylogenetic trees from molecular data. II. Gene frequency data. J Mol Evol.

[38] Sneath PHA, Sokal RR. Numerical taxonomy: The principles and practice of numerical classification. Hardcover edn. United States: Freeman & Company Limited, W H; 1973.

[39] Saitou N, Nei M (1987). The neighbor-joining method: a new method for reconstructing phylogenetic trees. Mol Biol Evol.

[40] Langella O. Populations: a population genetic software. In*:* vol. CNRS UPR9034., 1.2.28 edn; 1999. http://www.mybiosoftware.com/populations-1-2-32-population-genetic-software.html.

[41] Page RD (1996). TreeView: an application to display phylogenetic trees on personal computers. Comput Appl Biosci.

[42] Nei M, Gojobori T (1986). Simple methods for estimating the numbers of synonymous and nonsynonymous nucleotide substitutions. Mol Biol Evol.

[43] Kumar S, Nei M, Dudley J, Tamura K (2008). MEGA: a biologist-centric software for evolutionary analysis of DNA and protein sequences. Brief Bioinform.

[44] Takeshima SN, Sarai A, Saitou N, Aida Y (2009). MHC class II DR classification based on antigen-binding groove natural selection. Biochem Biophys Res Commun.

[45] Clutton-Brock J (1999). A natural history of domesticated mammals. 2nd edition edn.

[46] Bradley DG, Magee DA. Genetics and domestic cattle origins. In: Documenting domestication: new genetic and archaeological paradigms. Zeder MA, Bradley DG, Emshwiller E, Smith BD. editors. 1 edn. U. S. A: University of California Press; 2006: 317–328.

[47] Magee DA, MacHugh DE, Edwards CJ (2014). Interrogation of modern and ancient genomes reveals the complex domestic history of cattle. Animal Frontiers.

[48] Klein J (1987). Origin of major histocompatibility complex polymorphism: the trans-species hypothesis. Hum Immunol.

[49] Sena L, Schneider MP, Brenig B, Honeycutt RL, Womack JE, Skow LC (2003). Polymorphisms in MHC-DRA and -DRB alleles of water buffalo (Bubalus bubalis) reveal different features from cattle DR alleles. Anim Genet.

[50] Mishra SK, Niranjan SK, Banerjee B, Dubey PK, Gonge DS, Mishra BP, Kataria RS (2016). High genetic diversity and distribution of Bubu-DQA alleles in swamp buffaloes (Bubalus bubalis carabanesis): identification of new Bubu-DQA loci and haplotypes. Immunogenetics.

[51] Hedrick PW, Thomson G (1983). Evidence for balancing selection at Hla. Genetics.

[52] Hughes AL, Nei M (1989). Nucleotide substitution at major histocompatibility complex class II loci: evidence for overdominant selection. Proc Natl Acad Sci U S A.

[53] Liron JP, Peral-Garcia P, Giovambattista G (2006). Genetic characterization of argentine and Bolivian creole cattle breeds assessed through microsatellites. J Hered.

[54] Liron JP, Bravi CM, Mirol PM, Peral-Garcia P, Giovambattista G (2006). African matrilineages in American creole cattle: evidence of two independent continental sources. Anim Genet.

[55] Porto Neto LR, Jonsson NN, D'Occhio MJ, Barendse W (2011). Molecular genetic approaches for identifying the basis of variation in resistance to tick infestation in cattle. Vet Parasitol.

[56] Mikko S, Spencer M, Morris B, Stabile S, Basu T, Stormont C, Andersson L. A comparative analysis of Mhc DRB3 polymorphism in the American bison (Bison bison). J Hered. 1997;88(6):499–503. 10.1093/oxfordjournals.jhered.a023144 9419889

[57] Russell GC, Marello KL, Gallagher A, McKeever DJ, Spooner RL. Amplification and sequencing of expressed DRB second exons from Bos indicus. Immunogenetics. 1994;39(6):432–36. 10.1007/BF00176162 7910589

[58] Sitte K, East IJ, Lavin MF, Jazwinska EC. Identification and characterization of new BoLA-DRB3 alleles by heteroduplex analysis and direct sequencing. Anim Genet. 1995;26(6):413–17. 10.1111/j.1365-2052.1995.tb02693.x 8572364

[59] Wang K, Sun D, Zhang Y. Sequencing of 15 new BoLA-DRB3 alleles. Int J Immunogenet. 2008;35(4–5):331–32. 10.1111/j.1744-313X.2008.00772.x 18643841

[60] Das DN, Sri Hari VG, Hatkar DN, Rengarajan K, Saravanan R, Suryanarayana VV, Murthy LK. Genetic diversity and population genetic analysis of bovine MHC class II DRB 3.2 locus in three Bos indicus cattle breeds of Southern India. Int J Immunogenet. 2012;39(6):508–19. 10.1111/j.1744-313X.2012.01126.x 22607523

